# Investigating the Effect of Staining Beverages on the Structural and Mechanical Integrity of Dental Composites Using Raman, Fourier Transform Infrared (FTIR) Spectroscopy, and Microhardness Analysis

**DOI:** 10.3390/medicina61040590

**Published:** 2025-03-25

**Authors:** Ramona Dumitrescu, Iasmina-Mădălina Anghel, Carmen Opris, Titus Vlase, Gabriela Vlase, Daniela Jumanca, Atena Galuscan, Nicoleta A. Toderas, Roxana Oancea, Ruxandra Sava-Rosianu, Octavia Balean

**Affiliations:** 1 Translational and Experimental Clinical Research Centre in Oral Health, Department of Preventive, Community Dentistry and Oral Health, University of Medicine and Pharmacy “Victor Babes”, 300040 Timisoara, Romania; dumitrescu.ramona@umft.ro (R.D.); jumanca.daniela@umft.ro (D.J.); galuscan.atena@umft.ro (A.G.); roancea@umft.ro (R.O.); balean.octavia@umft.ro (O.B.); 2 Clinic of Preventive, Community Dentistry and Oral Health, “Victor Babes” University of Medicine and Pharmacy, Eftimie Murgu Sq. No. 2, 300041 Timisoara, Romania; 3 Department of Materials Engineering and Manufacturing, Faculty of Mechanics, Politehnica University, 300222 Timisoara, Romania; iasmina.anghel@student.upt.ro (I.-M.A.); carmen.opris@upt.ro (C.O.); 4 Research Centre for Thermal Analysis in Environmental Problems-ICAM, West University of Timisoara, Pestalozzi Street 16, 300115 Timisoara, Romania; titus.vlase@e-uvt.ro (T.V.); gabriela.vlase@e-uvt.ro (G.V.); 5 Department of Psychology, Faculty of Sociology and Psychology, West University of Timisoara, 300223 Timisoara, Romania; nicoleta.toderas01@e-uvt.ro

**Keywords:** dental composite resin, Raman spectroscopy, Fourier transform infrared (FTIR) spectroscopy, Vickers hardness, acidic beverage exposure, surface degradation

## Abstract

*Background and Objectives*: Resin composites are widely used in restorative dentistry due to their favorable mechanical properties and aesthetics. However, their clinical performance depends on factors such as chemical composition, microhardness, and exposure to environmental challenges like acidic beverages. *Materials and Methods*: This study evaluated the effects of coffee, red wine, and Coca-Cola on the surface microhardness and chemical composition of two resin composites (G-ænial A’CHORD and Omnichroma). A total of 40 disk-shaped specimens (20 per composite) were fabricated and divided into four groups: control, red wine, coffee, and Coca-Cola. Specimens were immersed in their respective solutions for 10 days, after which Raman spectroscopy, Fourier Transform Infrared (FTIR) spectroscopy and Vickers microhardness testing were conducted to assess mechanical and chemical changes. Paired t-tests were performed to compare pre- and post-immersion values, with significance set at *p* < 0.05. Raman and FTIR spectroscopy confirmed structural changes, including peak shifts and intensity variations, particularly in polymer–matrix interactions and filler compositions. *Results:* The Vickers microhardness test revealed significant reductions in hardness for both composites after immersion, with Omnichroma showing a greater susceptibility to softening than G-ænial A’CHORD (*p* < 0.01). Red wine and coffee immersion resulted in the most significant decreases, indicating the strong impact of polyphenolic and acidic interactions on the composite structure. *Conclusions*: These findings indicate that prolonged exposure to acidic and staining beverages compromises the mechanical and chemical integrity of resin composites, with Omnichroma being more affected than G-ænial A’CHORD. The findings suggest that the selection of composite materials should consider resistance to staining agents, especially in high-risk oral environments.

## 1. Introduction

In restorative dentistry, resin composites are growing in popularity due to their excellent mechanical qualities and superior aesthetic results. The various chemical compositions and mechanical characteristics of resin composites suggest that a wide range of parameters affect how well they work in clinical settings [1]. Strength, hardness, and resistance to wear or deformation are just a few of the universal or standardized attributes that are determined by a large portion of dental resin composite testing. Recent reviews have examined appropriate test methods for these crucial features. However, it is widely acknowledged that the clinician’s skill in manipulating the materials determines the highest amount of these qualities [2].

One of the most crucial factors taken into account while restoring the posterior teeth, which are under increased stress, is the surface microhardness of restorative materials. Composite resins with lower surface hardness are more prone to wear and plaque retention, increasing the risk of secondary caries, patient discomfort, and discoloration [3]. Over time, the biodegradation of resin composite may be accelerated by the crucial oral environment conditions, such as pH variations and humidity. Filler–polymer matrix debonding can be caused by pH and humidity both dissolving the polymer matrix; the release of leftover monomers eventually leads to early wear and erosion of composite restorations. Saliva with a low pH is more likely to experience polymer erosion, which can lead to discoloration, plaque buildup, and secondary caries [4]. The degradation of resin composites is significantly influenced by acidic conditions, as low pH environments have been shown to accelerate hydrolytic breakdown of the polymer matrix, weaken filler–matrix bonds, and increase surface roughness. Studies indicate that prolonged exposure to acidic beverages promotes material softening and structural erosion, ultimately compromising mechanical integrity and aesthetic longevity. Given the frequent consumption of such beverages, understanding the impact of pH on composite restorations is crucial for their long-term clinical performance [5,6].

It is thought to be essential for these restorations to function well inside the mouth for a long time. Therefore, in addition to caries, there are other types of damaging processes that can permanently alter the tooth surface in the oral environment which are also known as erosion, resorption, abrasion, and abfraction [7]. When exposed to organic acids and other food and beverage ingredients, resin-based composite filler materials are susceptible to weakening. During regular eating and drinking, these components often come into contact with a variety of chemical substances, such as alcohols, acids, salts, and alkalis. Moreover, the frequency of exposure to these compounds has a substantial impact on their deterioration. The composite’s microhardness, a crucial attribute that directly affects important physicochemical properties like compressive strength and abrasion resistance, may change as a result of this exposure. These alterations thus shorten the restoration’s lifespan, accelerate its degradation, and increase the need for replacement [8]. A previous literature review [3] analyzed the effects of various staining and acidic solutions, including coffee, tea, red wine, cola-based drinks, fruit juices, energy drinks, and chlorhexidine, on the color stability and surface roughness of resin composites, with a primary focus on nanohybrid, microhybrid, and bulk-fill composites. However, limited research has comprehensively assessed both the chemical and mechanical integrity of these materials using advanced spectroscopic techniques, highlighting the need for further investigation into their long-term stability in acidic and staining environments.

According to published research, continuous exposure to low pH beverages including cola and fruit juices (orange and apple cider), coffee, and beer can cause substantial surface alterations on tooth enamel and dental restorative materials. They mostly consist of changes in surface characteristics and microhardness [9].

Surface hardness, which is correlated with compressive strength, wear resistance, and resistance to intraoral softening, is one of the most crucial characteristics of a restorative material. In addition to making a surface more prone to wear and scratches, low surface hardness also reduces fatigue strength, which can result in restoration failure. A restorative material’s clinical performance and endurance are determined by its resistance to or limited display of changes in the oral cavity’s constantly shifting environment, which is caused by thermal, chemical, and mechanical forces [10].

Every molecule in a chemical composition vibrates continuously, and they can all increase their vibrational motion by absorbing energy from incoming photons. Raman spectroscopy and infrared (IR) spectroscopy are the two main vibrational spectroscopy techniques used to investigate these phenomena. A net change in the dipole moment during vibration or in the functional group under study is required for a molecule to absorb infrared radiation. Raman spectroscopy, on the other hand, has different selection criteria since Raman activity only happens when the vibration results in a net change in the polarizability of the molecule’s bonds [11]. This method can be used to determine the molecules’ chemical structure, bonding, conformation, and intermolecular interactions. Despite its similarities to infrared spectroscopy, the fundamental idea behind Raman spectroscopy is inelastic light scattering [2]. Raman spectroscopy enables multiple nondestructive measurements and minimum sample preparation, allowing specimens to be studied exactly as they are. Raman depends on inelastic scattering, as opposed to FTIR, which assesses the absorption of incoming radiation. After molecules are excited to a virtual energy level by laser light, they relax to a vibrational state and release photons with changed energy. Anti-Stokes–Raman scattering involves lower vibrational energy states, whereas Stokes–Raman scattering happens when photons are released at higher vibrational energy states. The vibrational energy states that are typical of particular functional groups and chemical bonds are reflected in the photon spectrum that is released [12].

This study builds on our previous research [13,14] by further evaluating the chemical and mechanical stability of these materials. Omnichroma, a single-shade universal composite, offers efficient shade matching but requires further study regarding its long-term resistance to degradation. Limited research is available on the mechanical, spectral, and structural properties of Omnichroma, with most studies focusing on its color adjustment potential [15,16], shade matching ability [17], optical behavior [18], color stability [19], cytotoxicity [20], as well as flexural strength (FS) and elastic modulus (EM) [21,22]. However, its resistance to chemical and mechanical degradation under prolonged exposure to acidic and staining environments remains insufficiently explored. Given its increasing clinical use as a single-shade universal composite, further investigation into its long-term performance is essential, which justifies its inclusion in this study. In contrast, G-ænial A’CHORD, a micro-hybrid composite, is recognized for its polishability, wear resistance, and aesthetics. However, despite its favorable mechanical and optical properties, its response to acidic and staining environments remains underexplored. Studies have shown that G-ænial A’CHORD exhibits superior microhardness and surface smoothness compared to other universal composites, which may influence its resistance to extrinsic staining and chemical degradation [23]. Additionally, its translucency and color stability have been assessed in controlled conditions, yet data on how exposure to acidic beverages affects its structural integrity are still lacking [24].

Previous studies have demonstrated that Omnichroma and G-ænial A’CHORD exhibit distinct mechanical and optical properties, which may influence their resistance to acidic and staining challenges. Research on Omnichroma has shown that color stability and surface roughness may be affected by staining agents and prolonged chemical exposure, leading to surface alterations [25]. Similarly, its optical properties have been reported to be influenced by aging processes [26]. G-ænial A’CHORD, on the other hand, has been studied for its microhardness and surface roughness, with findings suggesting that thermal changes and environmental conditions may impact its long-term performance [27]. Given these existing findings, our study expands upon previous research by evaluating how acidic and staining beverages affect the structural and chemical integrity of these composites, further supporting the role of material selection in clinical durability and aesthetic longevity.

Given the importance of surface hardness and chemical stability in determining the longevity of composite restorations, further investigation is necessary to determine how G-ænial A’CHORD performs in real-world conditions when subjected to acidic and staining beverages. This study examines how their filler composition, monomer structure, and polymer network stability affect their performance in acidic and staining environments, providing clinically relevant insights for material selection and long-term restorative success.

### 1.1. Study Aims

This study aims to evaluate the structural and mechanical integrity of two modern resin-based composite materials (G-ænial A’CHORD and Omnichroma) when exposed to commonly consumed acidic and staining beverages. Fourier Transform Infrared (FTIR) spectroscopy, Raman spectroscopy, and Vickers microhardness testing were employed to assess molecular and surface integrity changes in dental composites after controlled immersion periods. Through these analyses, the study examines the impact of prolonged exposure to coffee, red wine, and Coca-Cola on the chemical structure, vibrational properties, and surface hardness of the tested materials. The findings will contribute to a better understanding of the long-term durability of composite restorations in challenging oral environments.

### 1.2. Hypotheses

To achieve these objectives, three null hypotheses were formulated:

1. The type of acidic beverage will not significantly affect the microhardness, Raman spectral characteristics, or FTIR properties of the tested composite resins before and after immersion;
2. No significant differences will be observed in the degradation patterns (microhardness and vibrational spectroscopy data) among the two tested composite materials after exposure to acidic beverages;
3. The pH of the acidic beverages will not have a significant effect on the microhardness, Raman spectral characteristics, or FTIR properties of the tested composite resins before and after immersion.

## 2. Materials and Methods

This in vitro study assessed two resin dental composites that are commercially accessible in Romania and come from various manufacturers. The restorative materials investigated in this study were a micro-hybrid resin composite, which is selected for its ease of handling and superior polishability (G-ænial A’CHORD, GC Corporation, Tokyo, Japan), and a monocolor composite, which is well-known for its single-shade universal application and adaptation to the surrounding tooth color (Omnichroma, Tokuyama Dental, Tokyo, Japan).

Table 1 lists the manufacturers, lot numbers, types, shades, composite structure (monomers, filler composition/size), load percentages of the filler, and brand names.

### 2.1. Specimen Preparation

To prepare the specimens, 40 disk-shaped samples were fabricated, with 20 samples per material. The sample size was determined to achieve statistically significant results while considering practical constraints related to material availability and testing equipment capacity. Based on an effect size of 0.8, derived from preliminary studies and similar research in dental materials [13,14], along with a statistical power of 0.80 and an alpha level of 0.05, a minimum of 12 samples per group was required. However, to enhance result robustness, 20 samples per group were included, resulting in a total of 40 specimens across the two material groups.

The specimens, 20 per composite, were fabricated using calibrated circular plexiglass molds, each with a diameter of 10 mm and a thickness of 2 mm. A clean glass slab was positioned beneath the mold to ensure material support and uniform condensation. The resin composites were injected into the molds and covered with Mylar strips to create a smooth surface while minimizing the formation of an oxygen-inhibited layer. To further enhance uniformity, a thin glass slide was placed over the Mylar strip and gently pressed to eliminate excess material before polymerization. Following this, the glass slide was removed before light-curing the specimens. Polymerization of the specimens was carried out using a light-curing device (Bluephase G2, Ivoclar Vivadent, Mississauga, ON, Canada) with a light intensity of 1200 mW/cm^2^. The light-curing tip was positioned at a distance of approximately 1 mm from the sample surface for uniform curing. Each side of the mold was light-cured for 40 s to ensure thorough polymerization. Light intensity was confirmed using a calibrated light meter. After curing, the specimens were carefully removed from the molds and visually inspected to ensure uniformity and absence of defects.

After each daily immersion period, specimens were rinsed thoroughly with distilled water to remove any residual staining agents and then gently dried using absorbent paper before being placed in fresh distilled water at 37 °C in an incubator (Cultura Incubator 220–240 V, Ivoclar Vivadent) until the next immersion cycle. To ensure consistency, control group samples were also stored in distilled water at 37 °C for the entire study duration, without being exposed to any acidic or staining solutions. This condition was chosen to simulate intraoral hydration, serving as a baseline reference for assessing the chemical and mechanical changes induced in the experimental groups. To further ensure consistency and standardization, all immersion solutions (coffee, red wine, and Coca-Cola) were maintained at 37 °C in the incubator throughout the study. This temperature was selected based on previous research protocols [3] that assess the chemical stability and staining effects of resin composites under controlled laboratory conditions, mimicking the physiological environment of the oral cavity. Maintaining a controlled hydration state was essential to prevent dehydration or excessive water absorption, which could artificially influence composite properties. Additionally, to ensure that any observed changes were solely due to the immersion treatments, specimens were not exposed to UV radiation or other environmental factors beyond the intended experimental setup. These standardized storage conditions allowed for a controlled and reproducible evaluation of the effects of acidic and staining beverages on the tested materials.

All specimens underwent polishing for 20 s with each Sof-Lex disc (3M ESPE Dental Products, St. Paul, Minnesota, MN, USA). This step was essential to achieve a uniform and clinically relevant surface finish, as polishing enhances the aesthetic properties, reduces plaque accumulation, and improves the long-term performance of composite resins, as supported by previous studies [30,31,32]. Successive discs of varying abrasiveness, including Coarse (100 μm), Medium (29 μm), Fine (14 μm), and Super Fine (8 μm), were utilized. The polishing process was carried out by a single operator using a low-speed hand device at 15,000 rpm. Uniform, dry, and intermittent pressure was applied following the method described by Gonulol and Yilmaz [33]. Each specimen received polishing with a new disc to avoid contamination or wear inconsistencies. After polishing, all specimens were subjected to ultrasonic cleaning in distilled water for 5 min, followed by rinsing, to remove any residual debris. These standardized procedures ensured uniformity in specimen preparation for subsequent testing and analysis.

### 2.2. Raman Spectroscopy

The vibrational characteristics of composite resins were evaluated before and after immersion in acidic liquids (coffee, red wine, and Coca-Cola) using the LabRAM Soleil™ Raman Microscope (HORIBA Scientific, Edison, NJ, USA), equipped with the LabSpec 6 Spectroscopy Suite for Raman spectroscopy analysis. The system operated with a 532 nm laser and featured QScan™ lightsheet confocal imaging, ensuring high spatial resolution, along with SmartSampling™ technology for ultrafast spectral imaging.

To enhance signal quality and reduce noise, measurements were acquired with four accumulations. A 5× magnification objective lens was employed to precisely focus the laser beam onto the sample surface, covering a spectral range of 200 to 3200 cm^−1^, enabling a comprehensive vibrational profile. To optimize spectral acquisition while preventing thermal degradation of the material, a neutral density (ND) filter set to 10% (8.9 mW) was applied to control laser intensity. The LabSpec 6 software ensured accuracy and reproducibility by facilitating data acquisition, processing, and spectral analysis. Each Raman spectrum was obtained as an average of 20 scans to enhance signal accuracy and minimize variability, ensuring reliable and reproducible measurements across all specimens. This advanced Raman system enabled detailed spectral characterization, providing insights into the structural and vibrational modifications of the composite resins following exposure to acidic environments.

The method, which is based on the Raman effect, gives information about the vibrations of the atoms in a crystal lattice without being intrusive or harmful. A monochromatic light beam striking a sample’s surface can cause a number of physical effects, including reflection, transmission within the material, absorption, and scattering in all directions. Elastic or Rayleigh scattering is used when the scattered light’s wavelength matches that of the incoming light; inelastic or Raman diffusion is used when the scattered light’s wavelength differs from the excitation one. In this instance, the spectrum of the scattered light contains essential physicochemical information about the sample under investigation because the frequency of the radiation that results from the interaction is shifted with respect to the initial one by a quantity equal to that of the material’s lattice vibrations [9].

### 2.3. Fourier Transform Infrared (FTIR) Spectroscopy 

For both the control and immersed composite samples, Fourier Transform Infrared (FTIR) spectroscopy was performed using an IRTracer-100 Fourier Transform Infrared Spectrophotometer (Shimadzu Corporation, Kyoto, Japan) with Spectrum IRTracer-AIM-9000 software (v.2.27) for data acquisition and analysis. This method enabled a comprehensive analysis of the chemical structures and interactions within the resin composites. FTIR spectra were collected in the range of 4000–400 cm^−1^ with a resolution of 4 cm^−1^, ensuring high accuracy and sensitivity in detecting molecular vibrations and functional group changes. The instrument utilized a diamond ATR (Attenuated Total Reflectance) accessory to facilitate non-destructive sampling and ensure reproducibility. The analysis was performed to identify structural modifications caused by immersion in staining solutions (coffee, red wine, and Coca-Cola) by comparing the spectra of the control and exposed samples. Distinct peaks corresponding to ester, carbonyl, and aromatic group vibrations were monitored to assess the impact of chemical interactions between the resin matrix and the staining agents. Data collection was performed after 20 recordings at a resolution of 4 cm^−1^, in the range of 4000–400 cm^−1^ on the Shimadzu IRTracer-100 FTIR spectrometer with ATR using AIM-9000 software.

### 2.4. Vickers Microhardness Test

**Figure 1:**
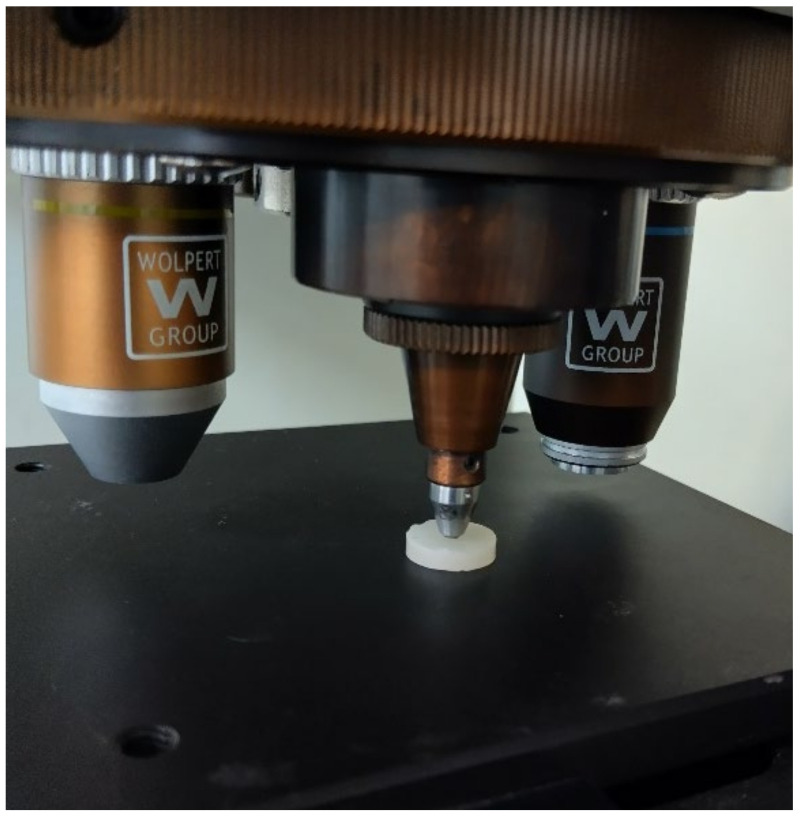
Specimen testing.

Microhardness was assessed using the Vickers method. The testing instrument, equipped with a diamond pyramid-shaped indenter, was configured with a load of 50 g and an indentation duration of 10 s. The sample was securely positioned during testing, and the indentation was performed under the specified parameters (Figure 1). 

**Figure 2:**
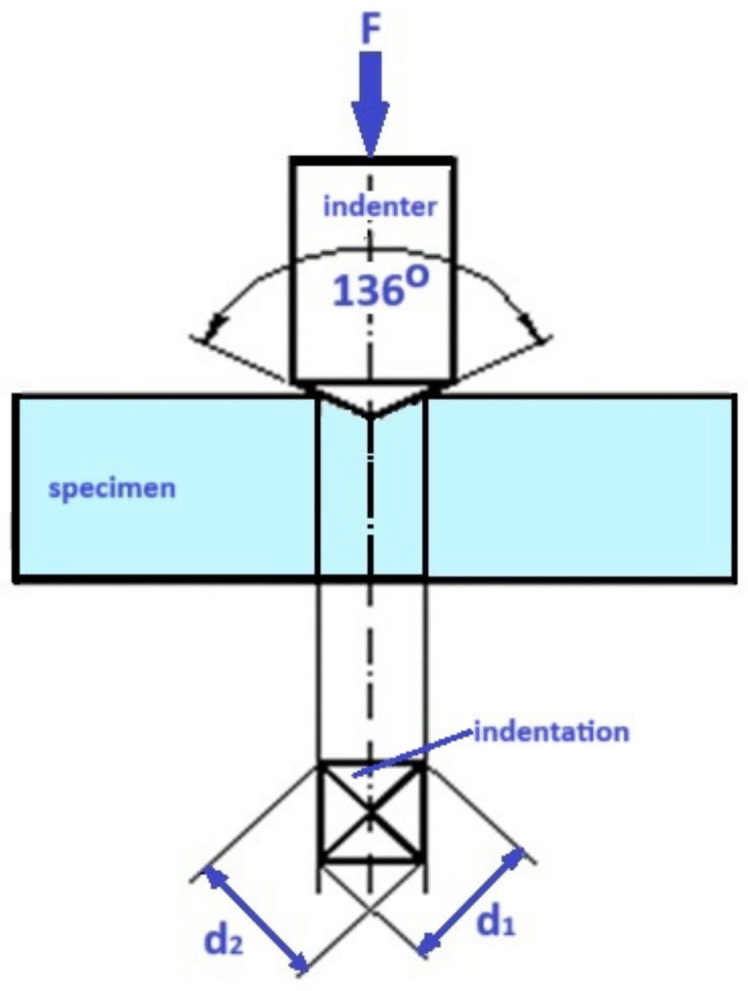
Schematics of the Vickers hardness test.

The equipment used for this investigation was a Wolpert Group Micro-Vickers Hardness Tester (Model: 402MVD). The results were recorded in terms of the Vickers Pyramid Number (HV), a standard unit of hardness measurement. The Vickers test is particularly advantageous compared to other hardness tests as its calculations are independent of the indenter size, allowing for its application across a wide range of materials regardless of hardness [34]. To determine Vickers microhardness values, the following formula was used:

Hv = 1.8544Pd/2

(Hv: Vickers microhardness, P: the indentation load, d: the length of the diagonal of the indentation) (Figure 2).

**Figure 3:**
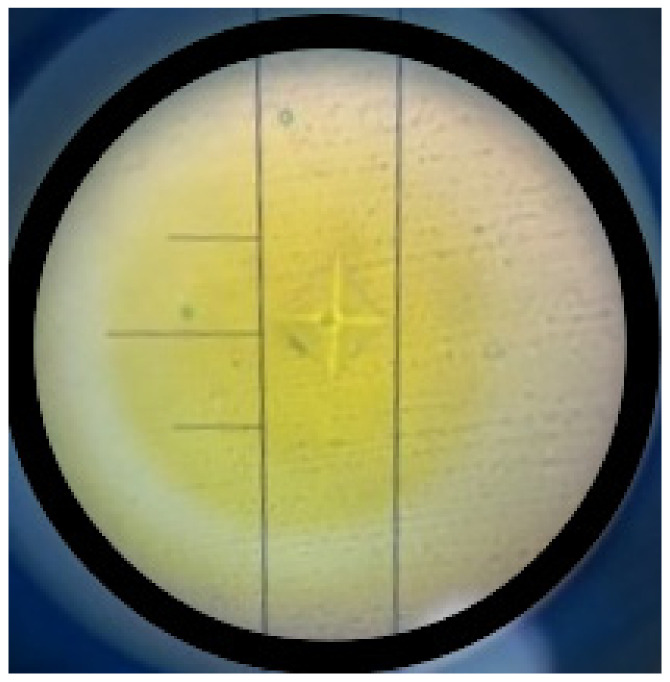
Vickers microhardness indentation.

To ensure measurement accuracy and account for potential surface variations, three indentations per specimen were performed at different locations, following standard Vickers microhardness testing protocols. The microhardness value for each specimen was calculated as the mean of these three measurements. This approach enhances the reliability of the results by minimizing localized inconsistencies and ensuring a representative assessment of each composite material’s surface hardness. Figure 3 presents an indentation mark obtained from the Vickers microhardness test, showing a well-defined impression on the specimen surface. The crosshair and calibration lines aid in accurately measuring the indentation’s diagonal length, essential for calculating the Vickers hardness value. The uniformity and clarity of the indentation reflect the material’s response to the applied load, providing insights into its mechanical properties. 

### 2.5. Immersion Protocol for Evaluating Beverage Adhesion to Composite Resins

Employing the two types of dental composite specimens, the adhesion of red wine, black coffee, and Coca-Cola to dental surfaces was investigated. These plates were immersed for 20 min every day for ten days in a row in black coffee that was made to standard infusion strength, 5 g to 150 mL of boiled water (Nespresso, Nestle Romania SRL, Bucharest, Romania), red wine (Budureasca Clasic, Feteasca Neagra, Dealu Mare, Romania), and Coca-Cola (The Coca-Cola Company, Atlanta, GA, USA). The three staining solutions used in this study were selected based on their common consumption and known effects on dental composites. Each beverage was freshly prepared or opened daily to prevent alterations in pH and chemical composition due to oxidation or carbonation loss, as reported in prior studies. For coffee, a standardized preparation protocol was followed, using 5 g of ground coffee brewed in 150 mL of boiling water. Red wine was stored in a sealed container at 4 °C when not in use and brought to room temperature before immersion. Coca-Cola was used directly from a freshly opened bottle to maintain carbonation and acidity levels. The pH of each solution was measured before each immersion session using a Milwaukee MW100 portable pH meter (Milwaukee Instruments, Loves Park, IL, USA) to ensure consistency across the experimental period. These handling procedures ensured that all immersion conditions closely reflected real-world scenarios while maintaining reproducibility in laboratory settings.

### 2.6. Determination of the pH Level

A Milwaukee MW100 portable pH meter was used to measure the staining solutions’ pH. This device delivered precise and reliable pH readings, which were essential for determining how acidic the study’s liquids were. Before the composite samples were submerged, the pH of each solution was adjusted to ensure uniform conditions for assessing the effect on the resin composites’ surface roughness and color stability. The electrode was cleaned with distilled water before each step, and the pH meter was calibrated using standard solutions prior to the measurement. The recorded pH values for the staining solutions were as follows: red wine (3.5), coffee (5.6), and Coca-Cola (2.4). These values indicate a range of acidity levels, from highly acidic (Coca-Cola and red wine) to moderately acidic (coffee), which could influence the chemical and mechanical properties of the tested composites.

### 2.7. Statistical Analysis

The pre-immersion and post-immersion data were used to calculate the percentage change, mean percentage change in microhardness, and standard deviation. Paired *t*-tests were performed to assess statistically significant differences in Vickers microhardness before and after immersion in staining beverages. The level of significance was set at *p* < 0.05, and statistical analysis was conducted using the Statistical Package for the Social Sciences (SPSS) software, version 23 (IBM, San Jose, CA, USA).

## 3. Results

### 3.1. Raman Analysis

**Figure 4:**
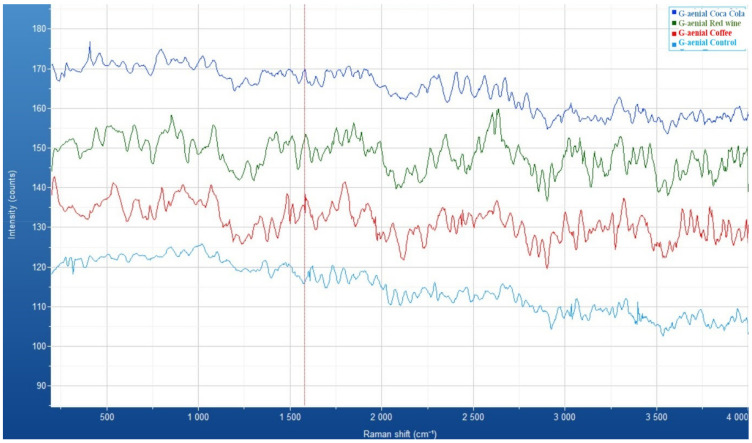
Raman spectra of G-ænial A’CHORD composite resins exposed to coffee (red), Coca-Cola (dark blue)), and red wine (green) and control (light blue).

The Raman spectra of the G-ænial A’CHORD composite resin before and after immersion in staining solutions (Coca-Cola, red wine, and coffee) reveal significant variations in vibrational characteristics, indicating potential chemical interactions with the acidic environments. The control sample exhibits distinct peaks within the 500–1800 cm^−1^ region, corresponding to C=C stretching (aromatic groups), C-H bending, and carbonyl (C=O) vibrations, while the 2800–3200 cm^−1^ range represents C-H stretching, characteristic of the polymeric matrix. Following immersion, the Coca-Cola-exposed sample shows increased intensity in the 1200–1700 cm^−1^ range, suggesting surface modifications rather than substantial degradation. The red wine-immersed sample displays intensity fluctuations and peak broadening, particularly in the 1000–1600 cm^−1^ range, likely due to interactions with tannins, anthocyanins, or acidic components. Similarly, the coffee-exposed sample exhibits peak broadening and intensity variations, especially in the C=O and C-H stretching regions, indicating mild structural modifications, potentially due to the adsorption of polyphenols and acids. Although these spectral changes suggest chemical interactions and surface adsorption of staining molecules, the absence of significant peak loss indicates that the structural integrity of the composite resin remains largely intact. Among the tested solutions, red wine and coffee induce more noticeable spectral variations compared to Coca-Cola, suggesting that these solutions have a greater potential to alter the material’s surface chemistry. These findings provide crucial insights into the impact of staining agents on the chemical stability, structural resilience, and long-term durability of the G-ænial A’CHORD composite resin (Figure 4).

**Figure 5:**
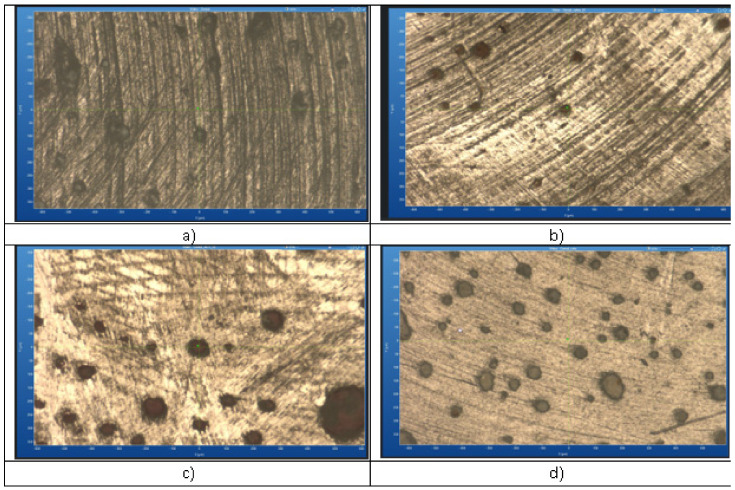
Microscopic Raman analysis of G-ænial A’CHORD composite: control (**a**), compared to effects of immersion in to coffee (**b**), red wine (**c**), Coca-Cola (**d**).

The microscopic Raman images (Figure 5) depict the surface morphology of the G-ænial A’CHORD composite after immersion in different staining beverages—red wine, Coca-Cola, and coffee—compared to the control sample. Each image reveals distinct changes in the composite’s surface texture and structural integrity, likely due to chemical interactions and staining effects. The red wine-treated sample exhibits pronounced dark deposits and a rougher surface, suggesting adsorption of pigments and potential matrix degradation. The Coca-Cola-immersed composite shows moderate surface alterations, with evidence of minor erosion likely caused by its acidic components. The coffee-treated sample presents a more uniform distribution of staining deposits, indicative of gradual discoloration rather than structural degradation. In contrast, the control sample maintains a relatively smooth and consistent surface, confirming the impact of beverage exposure on the composite’s microstructure.

**Figure 6:**
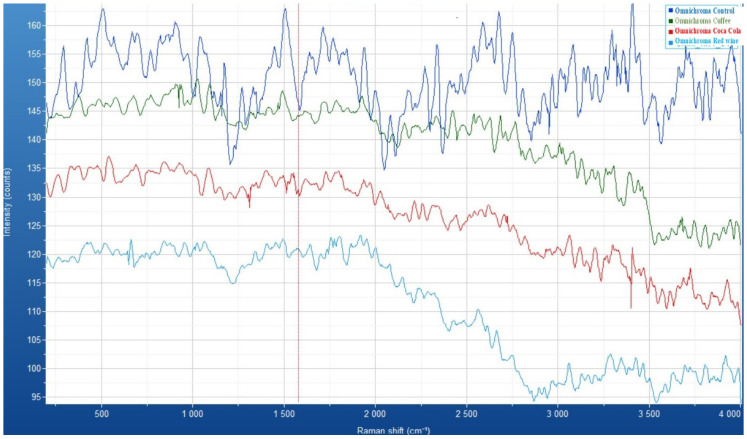
Raman spectra of Omnichroma composite: comparative analysis of control and samples immersed in coffee, Coca-Cola, and red wine.

For the Omnichroma resin composite, the Raman spectra illustrate spectral variations that suggest immersion in different beverages induces molecular and surface alterations in the material. The *x*-axis represents the Raman shift (cm^−1^), indicating vibrational molecular changes, while the *y*-axis denotes intensity (counts), corresponding to the scattered light signal. The control sample exhibits the highest intensity and well-defined peaks, while the spectra of the immersed samples reveal varying degrees of attenuation and structural modifications. The coffee- and wine-immersed samples demonstrate significant spectral fluctuations, likely due to chemical interactions or adsorption of staining agents. The Coca-Cola-treated sample shows a progressive reduction in intensity, suggesting possible surface degradation or penetration of acidic components into the composite matrix (Figure 6).

**Figure 7:**
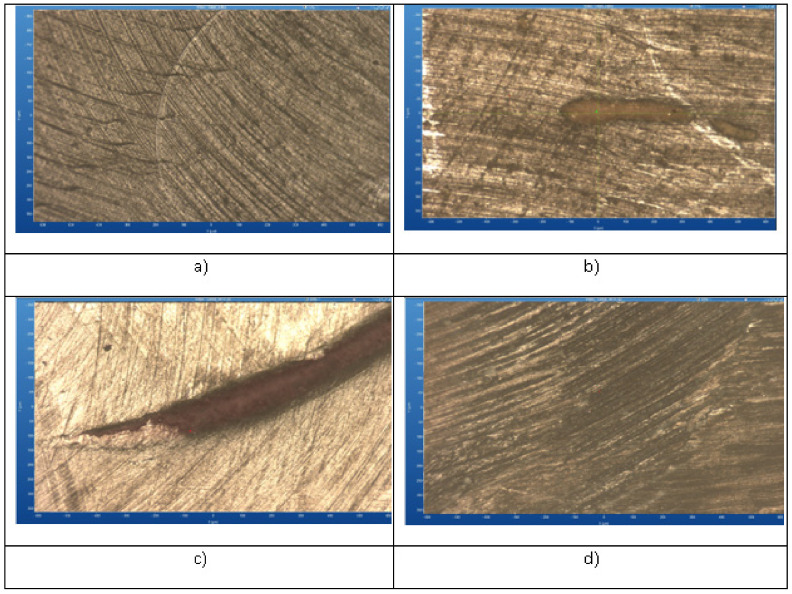
Microscopic analysis of Omnichroma composite: control (**a**), compared to effects of immersion in to coffee (**b**), red wine (**c**), Coca-Cola (**d**).

Figure 7 illustrates the surface morphology of the Omnichroma composite after exposure to different staining beverages—Coca-Cola, coffee, and red wine—compared to a control sample. Each image reveals distinct changes in surface texture, indicative of potential chemical interactions, staining, and material degradation. The Coca-Cola-treated sample exhibits visible surface irregularities and minor erosion, likely due to the acidic nature of the beverage. The coffee-immersed composite shows moderate staining with dispersed dark deposits, suggesting the adsorption of colorants onto the composite matrix. The red wine-exposed sample presents more pronounced staining with a deeper penetration of pigments along surface grooves, indicating strong interaction with the composite material. In contrast, the control sample maintains a smooth and uniform surface, with minimal alterations, confirming the impact of beverage exposure on the composite’s integrity.

**Figure 8:**
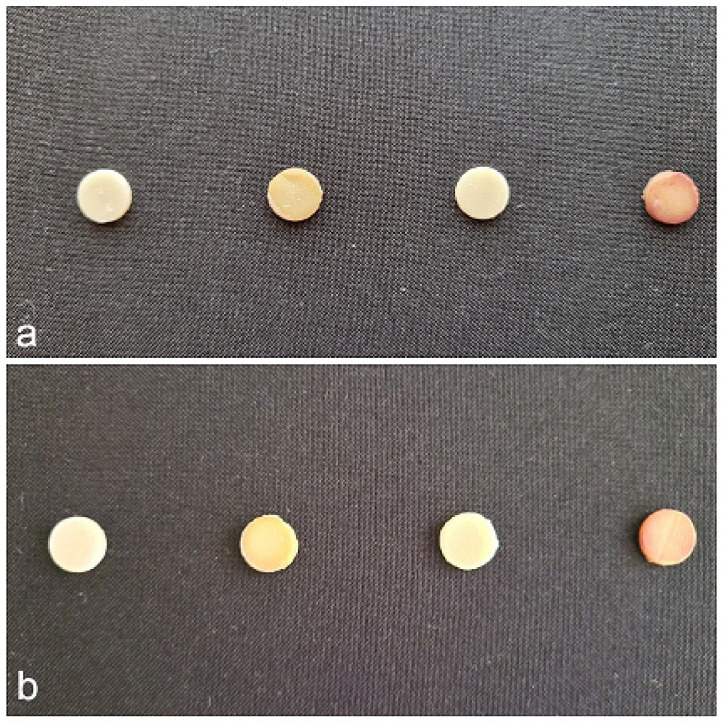
Macrophotographs of composite resin samples of Omnichroma (**a**) and G-ænial A’CHORD (**b**), control and after immersion in coffee, Coca-Cola, and red wine.

To further illustrate the effects of immersion in staining solutions, macrophotographs of the samples were captured before and after the 10-day exposure period. These images provide a visual representation of surface discoloration and potential material degradation, highlighting the varying degrees of color alteration among the tested composites. The differences in staining intensity and surface texture observed across the samples support the spectroscopic and microhardness findings, reinforcing the impact of acidic and polyphenolic agents on composite integrity (Figure 8).

### 3.2. FTIR Spectroscopy Analysis

**Figure 9:**
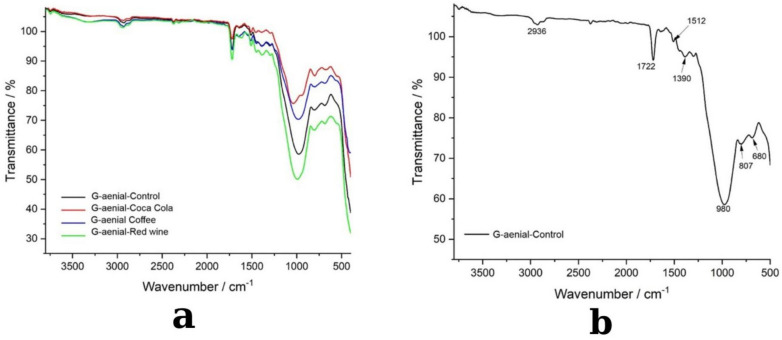
FTIR spectra of G-ænial A’CHORD composite samples exposed to different beverages: (**a**) transmittance spectra comparison, (**b**) detailed spectral peaks for the control sample.

The FTIR analysis of G-ænial A’CHORD composite resin before and after immersion in coffee, red wine, and Coca-Cola revealed distinct chemical modifications induced by each staining solution (Figure 9a,b).

Figure 9b presents the FTIR spectrum of the control sample, serving as a baseline for the composite’s chemical structure before exposure. The spectrum exhibits characteristic peaks corresponding to the polymer matrix and filler materials. The broad absorption band at 2936 cm^−1^ is associated with C-H stretching vibrations from aliphatic and aromatic hydrocarbons, reflecting the presence of Bis-GMA and UDMA monomers. The peak at 1722 cm^−1^ corresponds to C=O (carbonyl) stretching, indicative of ester groups that contribute to the polymer’s cross-linked network stability. Additionally, the 1512 cm^−1^ peak is assigned to aromatic C=C stretching, confirming the presence of rigid monomeric structures that enhance mechanical strength and resistance to deformation. The peaks at 1390 cm^−1^, 980 cm^−1^, 807 cm^−1^, and 680 cm^−1^ correspond to C-H deformation, C-O stretching, and Si-O-Si vibrations, which confirm the presence of silica-based inorganic fillers essential for improving wear resistance and durability. The well-defined peaks in the control sample suggest a stable molecular structure with a well-integrated filler–polymer interface, providing a reference for evaluating structural modifications induced by staining solutions.

Following immersion in staining agents, Figure 8a highlights substantial chemical alterations, particularly in the coffee-exposed spectrum (blue), which exhibits the most pronounced changes within the 1000–1700 cm^−1^ region. Notable shifts in carbonyl (C=O), aromatic (C=C), and silane coupling (Si-O-Si) groups suggest polymer degradation, ester hydrolysis, and disruption of the filler–matrix interface, which may contribute to mechanical instability and surface softening. These findings indicate strong interactions between polyphenol-rich compounds in coffee and the polymer network, leading to progressive molecular breakdown. The red wine-exposed spectrum (green) shows moderate structural alterations, particularly in the 900–1400 cm^−1^ region, indicating polyphenolic interactions with ester functional groups, potentially causing cross-linking disruptions and polymer rearrangement. Conversely, the Coca-Cola-exposed spectrum (red) exhibits minimal variations, with only minor shifts near 1700 cm^−1^ and 1200 cm^−1^, indicating limited hydrolytic degradation due to acidic exposure but without significant structural compromise.

Overall, the FTIR results (Figure 9a,b) confirm that coffee induces the most significant chemical modifications, followed by red wine, while Coca-Cola has the least effect on the structural integrity of G-ænial A’CHORD composite resin. 

**Figure 10:**
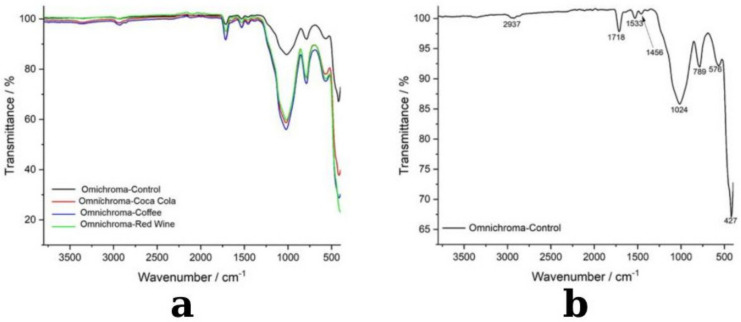
FTIR spectra of Omnichroma composite samples exposed to different beverages: (**a**) transmittance spectra comparison, (**b**) detailed spectral peaks for the control sample.

The FTIR analysis of Omnichroma composite resin before and after immersion in red wine, coffee, and Coca-Cola revealed distinct chemical modifications induced by each staining solution, with varying degrees of degradation and structural changes (Figure 10b). These results provide insights into the effects of dietary staining agents on the molecular stability and integrity of dental composite materials.

Figure 10b presents the FTIR spectrum of the control Omnichroma sample, serving as a reference for its chemical composition before exposure to staining solutions. The spectrum displays characteristic absorption peaks associated with the polymer matrix and filler components, confirming the presence of essential structural elements that contribute to the composite’s mechanical performance. The broad absorption band at 2937 cm^−1^ corresponds to C-H stretching vibrations, which are indicative of the resin’s organic backbone, primarily composed of UDMA and TEGDMA monomers. The strong peak at 1718 cm^−1^ is assigned to C=O (carbonyl) stretching, representing ester functional groups that play a crucial role in maintaining the cross-linked polymer network and overall structural integrity of the material. A distinct peak at 1533 cm^−1^ corresponds to C=C stretching in aromatic rings, confirming the presence of rigid monomer structures that enhance mechanical strength, wear resistance, and dimensional stability. Additionally, the 1456 cm^−1^ peak represents C-H bending vibrations, primarily associated with methylene (-CH_2_-) groups, which contribute to the flexibility and toughness of the polymer network. The peak at 1024 cm^−1^ is attributed to C-O stretching, a functional group essential for polymer backbone stability and intermolecular bonding. The observed peaks at 789 cm^−1^ and 576 cm^−1^ correspond to Si-O-Si stretching vibrations, confirming the presence of silica-based fillers, which are crucial for reinforcing mechanical properties and enhancing wear resistance. Finally, the low-frequency peak at 427 cm^−1^ is associated with inorganic filler-related vibrations, further validating the structural reinforcement properties of the composite. The well-defined peaks in the control sample indicate a stable molecular structure with a well-integrated filler–polymer interface, providing a critical reference point for assessing chemical modifications and degradation patterns following exposure to staining solutions.

Following immersion in dietary staining agents, Figure 10a highlights substantial chemical modifications, with red wine and coffee inducing the most pronounced spectral alterations. The red wine-exposed spectrum (green) exhibits significant deviations in the 900–1600 cm^−1^ region, where notable shifts in C=O (carbonyl), C=C (aromatic), and Si–O–Si (silane coupling) functional groups suggest polymer degradation, ester hydrolysis, and filler–matrix interface weakening. These modifications indicate strong chemical interactions between polyphenols in red wine and the composite resin, leading to molecular rearrangement and potential reductions in mechanical integrity. Similarly, the coffee-exposed spectrum (blue) demonstrates substantial changes, particularly in the 1000–1700 cm^−1^ range, where shifts in carbonyl, aromatic, and siloxane bonds suggest chemical softening and depolymerization of the resin matrix. These findings indicate that tannins and acidic components in coffee interact with the polymer network, potentially affecting long-term stability. In contrast, the Coca-Cola-exposed spectrum (red) displays less pronounced spectral variations, with only minor transmittance shifts around 1700 cm^−1^ and 1200 cm^−1^, indicating limited hydrolytic degradation due to acidity but without substantial polymer disintegration.

Within the FTIR spectra, the changes suffered by the material do not only lead to the absence or appearance of vibrations in the FTIR spectrum of the materials. What is observed within the spectra is only a change in the intensity of the peaks, which leads us to the conclusion of a change on its surface. Changes are observed in the range of 1000–1700 cm^−1^ because in the case of red wine, the change in the range of 1600-1500 cm^−1^ can be argued to occur by the binding of polyphenols in the composition of the wine, in the case of coffee, an effect on the ester bond of the basicity given by coffee can be suspected, and in the case of Coca-Cola, being acidic, it generates protons that bind to the C-O bond leading to the obtaining of C-OH. These changes are not total and therefore the changes are only partial.

### 3.3. Vickers Microhardness Analysis

The microhardness of the studied composite materials was measured using the Vickers method. The results for each material, including individual measurements and their average values, are summarized below. The obtained hardness values are expressed in terms of the Vickers Pyramid Number (HV) (Table 2).

The Vickers hardness of two resin composite materials, G-ænial A’CHORD and Omnichroma, was analyzed to evaluate the effects of submersion in red wine, coffee, and Coca-Cola over a 10-day period compared to control samples. The results demonstrate a consistent trend of reduced hardness for all materials after exposure to the test solutions. Similarly, G-ænial A’CHORD exhibited a mean hardness of 31.00 (±0.84) in the control group, which declined to 29.20 (±0.96) in red wine, 28.4 (±1.14) in coffee, and 28.30 (±1.30) in Coca-Cola. For Omnichroma, the control group’s mean hardness was 27.44 (±1.53), which significantly decreased to 29.6 (±0.58), 27.44 (±0.84), and 30.26 (±0.73) following immersion in red wine, coffee, and Coca-Cola, respectively. 

Statistical analysis using paired *t*-tests demonstrated significant differences in Vickers hardness for both Omnichroma and G-ænial A’CHORD composites after immersion in staining solutions. For Omnichroma, the most significant decrease was observed after red wine immersion (t(18) = 8.916, *p* < 0.01), followed by coffee (t(18) = 8.005, *p* < 0.01) and Coca-Cola (t(18) = 4.300, *p* < 0.01), indicating a notable reduction in hardness. Similarly, G-ænial A’CHORD composite showed a significant hardness decrease, though less pronounced than Omnichroma, with the highest impact from red wine (t(18) = 4.001, *p* < 0.01), followed by coffee (t(18) = 3.520, *p* < 0.01) and Coca-Cola (t(18) = 3.168, *p* < 0.01). These results suggest that both composites undergo mechanical degradation after prolonged exposure to staining agents, with Omnichroma being more susceptible to softening than G-ænial A’CHORD.

## 4. Discussion

Adequate mechanical, biological, and physical qualities against the abrasive and erosive oral environment are necessary for resin-based composite restorations to be successful [35]. This study aimed to evaluate the structural and mechanical integrity of two modern composite resins by analyzing changes in surface microhardness, Raman spectra, and FTIR characteristics following immersion in three acidic and staining beverages.

This study’s Raman spectroscopy investigation revealed material-specific and solution-specific interactions, showing that composite resins undergo detectable chemical changes when exposed to acidic and polyphenol-rich staining solutions. The results imply that staining solutions can change the chemical composition of dental composites, especially those that are strong in acidity (Coca-Cola) or polyphenolic chemicals (red wine). Despite being less acidic, coffee still contributed significantly to spectral fluctuations, especially in the organic matrix. Due to its distinct monocolor resin composition and filler structure, Omnichroma showed the most noticeable spectral and microhardness fluctuations among the investigated materials. This could potentially increase its sensitivity to degradation. G-ænial A’CHORD, on the other hand, showed more stability; however, its reaction differed based on the solution, suggesting that it was resistant to chemical interactions differently. Staining agents may be able to permeate the composite surface and cause molecular changes in ester, carbonyl, and aromatic structures, according to the observed peak shifts and intensity variations in Raman spectra. These findings emphasize how crucial it is to choose composite materials with improved resistance to chemical deterioration, particularly in clinical settings with frequent exposure to acidic and staining liquids.

In order to improve composite formulations’ resistance to mechanical and chemical deterioration in oral environments, future studies should examine long-term stability and optimization.

This study’s FTIR research showed that staining solutions cause chemical changes in composite resins that are particular to the material. The carbonyl (C=O), aromatic (C=C), and silane coupling (Si–O–Si) functional groups in coffee and red wine, in particular, saw the most notable changes among the examined beverages, indicating matrix softening, ester hydrolysis, and polymer degradation. The most noticeable spectrum changes were seen in the monocolor composite, suggesting a greater vulnerability to interactions with acidic and polyphenolic substances, which could eventually result in more structural instability. The hybrid composite, on the other hand, demonstrated increased resistance, less spectrum shifts, and milder degradation effects, so confirming the protective function of polymer cross-linking and filler composition. Both composites showed fewer structural changes in response to Coca-Cola, a predominantly acidic agent, than in response to coffee and red wine, indicating that polyphenols contribute more to degradation than acidity alone. Nevertheless, because of its unique filler–polymer interactions, the monocolor composite nevertheless displayed wider peak shifts, suggesting a greater vulnerability to hydrolytic processes. These results imply that chemical resistance is greatly influenced by composite composition, with hybrid composites showing increased stability against exposure to acid and food stains. In order to improve long-term clinical performance in restorations that are regularly exposed to staining drinks, future research should concentrate on creating more resistant formulations. These findings underscore the role of polyphenolic and acidic agents in composite degradation and emphasize the need for developing restorative materials with enhanced resistance to staining and acidic beverages, particularly in clinical settings where restorations are frequently exposed to dietary challenges.

Hardness, determined by the material’s microstructure and composition, denotes its capacity to withstand persistent deformation, commonly assessed via an indentation test. In this study, the surface hardness of the tested composite resin was measured using the Vickers hardness test, chosen for its simplicity and the reliability of the data it provides.

Vickers hardness values of dental composites typically range from 30 to over 100. However, to effectively mimic natural tooth tissues, a VHN value of 40–50 is recommended [36]. Microhardness is influenced by several factors, including filler type, morphology, and particle size, with higher filler content generally leading to increased hardness [37,38]. Our results indicate that exposure to staining agents significantly affects the surface microhardness of composite resins, with notable differences between the two tested materials. Omnichroma exhibited greater susceptibility to hardness reduction, particularly after immersion in red wine and coffee, indicating that polyphenolic compounds and acidity play a critical role in composite degradation. In contrast, G-ænial A’CHORD showed higher resistance, particularly after Coca-Cola immersion, suggesting that its filler composition and polymer matrix contribute to better mechanical stability. Across all tested conditions, control samples consistently exhibited the highest hardness values, confirming that the materials maintain their optimal mechanical properties in the absence of external chemical influences. While all tested composites experienced a decrease in microhardness after 10 days of immersion, the extent of this reduction was material-dependent, reinforcing the importance of composite formulation in determining long-term resistance to staining and acidic environments. These findings concurred with those of Al-Shekhli and Aubi [39] and Khan et al. [40]. 

The study’s results disprove all three null hypotheses stated in the Introduction. The first null hypothesis, which proposed that immersion in acidic beverages would not alter the tested composites’ microhardness, Raman spectral features, or FTIR properties, was refuted by significant mechanical and chemical changes observed post-immersion. Notable decreases in Vickers microhardness, particularly in the monocolor composite, and shifts in Raman and FTIR spectra confirmed that both acidity and polyphenolic interactions contributed to degradation. The second null hypothesis, suggesting that no significant differences would be observed in the degradation patterns between the two composites, was also rejected. The results demonstrated that Omnichroma exhibited more pronounced hardness loss and spectral shifts, indicating greater susceptibility to chemical and mechanical degradation, whereas G-ænial A’CHORD showed comparatively higher stability. Finally, the third null hypothesis, which stated that the pH of the acidic beverages would not significantly affect the degradation of the composites, was contradicted by the findings. Coca-Cola, which had the lowest pH, induced significant hardness loss but exhibited fewer FTIR spectral changes, whereas coffee and red wine, despite being less acidic, caused more pronounced molecular degradation, highlighting the role of both acidity and polyphenolic content in composite deterioration. These findings reinforce the importance of material selection in restorative dentistry, particularly in environments with frequent exposure to acidic and staining beverages.

According to Voltarelli et al. [41], a material’s microhardness is directly linked to its resistance to intra-oral softening, compressive strength, and degree of conversion. Therefore, in this study, changes in microhardness were assessed to evaluate the degradation of dental materials subjected to exposure to various beverages. According to Abu-Bakr et al. [42], the surface of composite resins is seriously harmed by various beverages with varying pH levels, and surface degradation is more pronounced at lower pH levels. The physical and mechanical properties of the composite may deteriorate due to chemical degradation brought on by filler–matrix debonding, hydrolytic degradation of the fillers, or hydrolytic breakdown of the binding between silane and the filler particles [4].

The degradation in the physical and mechanical properties of restorative materials could be attributed to variations in the composition of immersion solutions. Factors such as the specific types of acids present, the pH level, and the concentration of acids in these solutions play a crucial role. These elements collectively contribute to the deterioration of surface hardness, primarily through the erosion and breakdown of the matrix/filler interface [39]. Numerous studies have demonstrated that exposure to low pH environments can lead to the detachment of fillers from resin materials and the degradation of the matrix component [39].

Because the polymer erodes at low salivary pH, composite resin is vulnerable to degradation. A shift in salivary pH and damage to the composite repair can be caused by a variety of causes, including eating habits and brushing behaviors. The oral cavity’s chemicals will cause restorations’ surfaces to become rougher and softer. The matrix of resin-based restorations is completely affected differently by various food-simulating organic solvents. For instance, organic acids found in many foods, beverages, and alcohol can weaken the resin matrix, and oral fluids can dissolve the coupling agent, whereas weak acids and water can harm inorganic filler [4].

Although there is a fair amount of studies on the microhardness of resin composites, it is challenging to compare them because of the wide variations in the experimental methodologies employed [43]. It is generally acknowledged that a resin composite’s mechanical behavior improves with increasing filler content, which in turn increases the restoration’s potential longevity [43]. Microhardness often denotes a material’s ability to withstand plastic deformation and abrasion wear. Increased crosslinking from polymerization events has a major impact on microhardness [1]. The chemical composition and mechanical properties of resin composites indicate that various factors impact their clinical performance [44]. Single-shade universal resin composites, with their unique color-matching mechanisms enabled by their filler structures, may demonstrate distinct mechanical and clinical behaviors [21]. The present study’s results underline the importance of material selection in clinical practice, particularly in cases where patients are likely to consume beverages such as coffee, soda, or wine.

In our study, the greater susceptibility of Omnichroma to degradation compared to G-ænial A’CHORD can be attributed to differences in their filler technology, monomer composition, and surface properties. Omnichroma incorporates supra-nano spherical fillers (260 nm), which, while designed for universal shade matching, may contribute to increased surface exposure to staining agents. In contrast, G-ænial A’CHORD utilizes a hybrid filler system, incorporating pre-polymerized resin fillers that enhance mechanical stability and stain resistance. Additionally, Omnichroma’s resin matrix, composed of UDMA and TEGDMA, may exhibit higher water sorption and staining susceptibility due to TEGDMA’s hydrophilic nature. Conversely, G-ænial A’CHORD, which employs a Bis-MEPP-based resin, may have a more hydrophobic matrix, offering greater resistance to discoloration and degradation. Moreover, differences in polymerization behavior and degree of conversion could further contribute to variations in material durability. The interaction of these factors likely explains the greater degradation observed in Omnichroma following exposure to acidic and staining beverages.

The pronounced degradation effects observed with red wine and coffee can be attributed to their high content of tannins and acidic components. Tannins, being high-molecular-weight polyphenolic compounds, have a strong affinity for proteins and minerals, enabling them to interact with the organic matrix of dental composites, leading to discoloration and potential structural changes. Additionally, the low pH of these beverages facilitates hydrolytic degradation of the ester bonds in the resin matrix, resulting in surface softening and erosion. The synergistic effect of tannins and acidity accelerates the degradation process, compromising both the mechanical properties and aesthetic qualities of the resin composites [45,46,47].

Future research should investigate the long-term effects of these exposures on composite materials, considering variables such as repeated exposure cycles and aging processes. Additionally, developing composites with enhanced resistance to acidic and staining agents could improve the longevity of dental restorations and ensure better patient satisfaction. These insights provide valuable guidance for clinicians in selecting the most suitable materials to achieve optimal mechanical performance and long-term outcomes under diverse conditions.

The clinical applicability of these findings is essential in guiding restorative strategies and patient education to enhance the longevity of composite restorations. Understanding the impact of acidic and staining beverages on composite materials allows clinicians to provide targeted recommendations. Patients should be educated on dietary habits, particularly regarding the potential effects of frequent exposure to these beverages. Encouraging proper oral hygiene practices, such as rinsing with water after consumption, can help mitigate surface degradation and staining. For individuals with high exposure to acidic or staining drinks, the use of protective coatings or surface sealants may serve as an additional barrier to enhance restoration durability. Furthermore, selecting composite materials with improved stain resistance and lower susceptibility to hydrolytic degradation is crucial, particularly in cases where both aesthetic outcomes and long-term performance are key considerations.

This study stands out due to its multimodal analytical technique that combines Raman spectroscopy, FTIR analysis, and Vickers microhardness testing to provide a thorough evaluation of both mechanical and molecular-level alterations in composite resins. The results are extremely useful to clinical dentistry since the 10-day immersion period using three regularly consumed beverages closely mimics real-world exposure. The findings emphasize the significance of composite selection for long-term repair durability by highlighting material-dependent variations in degradation. For dentists looking to improve restoration longevity and patient outcomes in high-risk oral environments, these findings are essential. 

This study builds upon previous research analyzing multiple composite resins, allowing for a more focused comparison of two distinct materials with increasing clinical relevance. Omnichroma, a single-shade composite, is widely used for its simplified shade matching and efficient handling, while G-ænial A’CHORD, a micro-hybrid composite, is valued for its balanced mechanical and aesthetic properties. The recent literature also supports studies that analyze two composite resins to ensure a controlled and meaningful evaluation. Unlike comparisons with other material classes, such as glass ionomers, this study aimed to assess performance differences within the same category, offering direct clinical implications

Despite the insights provided by this study, several limitations should be acknowledged. As an in vitro study, it does not fully replicate the complex intraoral environment, where factors such as salivary enzymes, mechanical forces from mastication, bacterial biofilm formation, and temperature fluctuations play a significant role in the degradation of composite resins. The absence of saliva simulation, which plays an essential role in the oral environment by buffering pH changes, influencing composite degradation, and affecting staining susceptibility, is one of our study limitations. The presence of enzymes, proteins, and minerals in saliva may interact with both composite materials and staining agents, potentially altering their effects. While our study focused on isolating the impact of acidic and staining beverages under controlled conditions, future research should incorporate artificial saliva to provide a more comprehensive assessment of composite behavior in simulated intraoral conditions. Additionally, the study did not incorporate thermocycling or artificial aging processes, which are commonly used to simulate the thermal and mechanical stresses that restorative materials undergo in the oral cavity. The 10-day immersion period, while useful for assessing early-stage material degradation, may not fully capture the effects of prolonged exposure to acidic and staining agents in clinical conditions. Future studies should explore extended immersion durations alongside thermocycling and artificial aging techniques to provide a more comprehensive understanding of long-term material durability. Another limitation is the selection of only two composite resins, chosen for their clinical relevance and distinct compositions. While this approach allowed for an in-depth comparison, the findings cannot be generalized to all resin composites, particularly those with significantly different filler compositions, polymer matrices, or curing mechanisms. Expanding the study to include bulk-fill composites, nanocomposites, and bioactive materials would offer a broader understanding of how various formulations respond to acidic and staining challenges. Furthermore, the study tested only three acidic beverages, which may not fully capture the diversity of dietary factors affecting composite restorations in daily use. Future research should explore a wider range of beverages with varying pH levels and chemical compositions, such as fruit juices, energy drinks, and alcoholic beverages, to better simulate real-world conditions. Despite these limitations, the controlled in vitro design ensured precise and reproducible results, allowing for a focused analysis of the chemical and mechanical changes in the selected composite materials. Addressing these limitations in future studies will help refine our understanding of composite material degradation and contribute to the development of more durable and clinically resilient restorative materials.

## 5. Conclusions

This study emphasizes how the molecular structure and mechanical integrity of composite resins are affected by acidic and staining beverages. After immersion, Raman spectroscopy showed notable changes in vibrational spectra, especially at the interfaces between the polymer matrix and filler, suggesting structural changes brought on by interactions with acidic agents and polyphenolic chemicals. These chemical interactions were further validated by FTIR spectroscopy, which revealed changes in functional group absorption, especially in ester and aromatic areas, indicating polymer degradation and possible filler–matrix bonding disruption. The monocolor composite was more prone to softening than the micro-hybrid composite, according to Vickers microhardness analysis, which showed statistically significant decreases in surface hardness after extended exposure. This further supports the importance of formulation and filler composition in long-term durability. These results highlight how crucial it is to take beverage-induced deterioration into account in clinical settings, especially when it comes to restorations in high-stress settings. Longer exposure times and other composite formulations should be examined in future research to better assess how staining and acidic agents affect mechanical performance and structural stability.

## Figures and Tables

**Table 1:** Specifications of the composite resins used in the study.

Product Name	Type	Composition	Lot Number	Manufacturer
G-ænial A’CHORD [28]	Hybrid resin composite	Filler: Pre-polimerized fillers containing silica, pre-polimerized particles containing strontium and lanthanoid fluride, silica, fumed silica. Base resin: Bis-MEPP-based resin.	230328C	Gc Tokyo, Japan
Omnichroma [29]	Supra-nanospherical resin composite	Filler: Uniform-sized supra-nano spherical filler (SiO_2_–ZrO_2_ 260 nm), round-shaped composite filler (containing 260 nm spherical SiO_2_–ZrO_2_). Base resin: UDMA, TEGDMA.	123E83	Tokuyama-Dental, Japan

**Table 2:** Descriptive statistics of Vickers microhardness for G-ænial A’CHORD and Omnichroma composites under different conditions.

Product Name		Mean (Standard Deviation)	Skewness	Kurtosis
Omnichroma	Control	27.44 (±1.53)	0.01	−1.14
	Red Wine	29.6 (±0.58)	0.91	1.92
	Coffee	27.44 (±0.84)	0.31	−0.68
	Coca-Cola	30.26 (±0.73)	−0.18	−1.58
G-ænial A’CHORD	Control	31.00 (±0.84)	0.07	−0.52
	Red Wine	29.20 (±0.96)	0.98	0.23
	Coffee	28.4 (±1.14)	0.79	−0.20
	Coca-Cola	28.30 (±1.30)	−0.00	−1.34

## Data Availability

The original contributions presented in this study are included in the article. Further inquiries can be directed to the corresponding author.
